# Left ventricular mechanoenergetics in excised, cross-circulated rat hearts under hypo-, normo-, and hyperthermic conditions

**DOI:** 10.1038/s41598-018-34666-3

**Published:** 2018-11-02

**Authors:** Koji Obata, Daisuke Takeshita, Hironobu Morita, Miyako Takaki

**Affiliations:** 1 Department of Physiology, Gifu University Graduate School of Medicine, 1-1 Yanagido, Gifu, 501-1194 Japan; 2 Department of Artificial Organs, National Cerebral and Cardiovascular Center Research Institute, Suita, 565-8565 Japan; 3 Department of Orthopaedic Surgery, Nara Medical University, School of Medicine, 840 Shijo-cho, Kashihara, Nara 634-8521 Japan

## Abstract

We investigated the effects of altering cardiac temperature on left ventricular (LV) myocardial mechanical work and energetics using the excised, cross-circulated rat heart model. We analyzed the LV end-systolic pressure–volume relationship (ESPVR) and linear relationship between myocardial oxygen consumption per beat (VO_2_) and systolic pressure–volume area (PVA; total mechanical energy per beat) in isovolumically contracting rat hearts during hypo- (32 °C), normo- (37 °C), and hyperthermia (42 °C) under a 300-beats per minute pacing. LV ESPVR shifted downward with increasing cardiac temperature. The VO_2_–PVA relationship was superimposable in these different thermal conditions; however, each data point of VO_2_–PVA shifted left-downward during increasing cardiac temperature on the superimposable VO_2_–PVA relationship line. VO_2_ for Ca^2+^ handling in excitation–contraction coupling decreased, which was associated with increasing cardiac temperature, during which sarcoplasmic reticulum Ca^2+^-ATPase (SERCA) activity was suppressed, due to phospholamban phosphorylation inhibition, and instead, O_2_ consumption for basal metabolism was increased. The O_2_ cost of LV contractility for Ca^2+^ also increased with increasing cardiac temperature. Logistic time constants evaluating LV relaxation time were significantly shortened with increasing cardiac temperature related to the acceleration of the detachment in cross-bridge (CB) cycling, indicating increased myosin ATPase activity. The results suggested that increasing cardiac temperature induced a negative inotropic action related to SERCA activity suppression in Ca^2+^ handling and increased myosin ATPase activity in CB cycling. We concluded that thermal intervention could modulate cardiac inotropism by changing CB cycling, Ca^2+^ handling, and basal metabolism in rat hearts.

## Introduction

The heart maintains its pumping action by converting chemical energy into mechanical work, which activates cross-bridge (CB) cycling that is composed of myosin and actin filaments via myosin ATPase. ATP is the chemical energy used for mechanical contraction, and most ATPs are produced by oxidative phosphorylation in the mitochondria. Calcium (Ca^2+^) is also a key role player in excitation–contraction (E-C) coupling and contributes to cardiac contractility. Myocardial temperature sensitively affects cardiac contractility and energy metabolism. Previous studies showed that hyperthermic intervention elicited negative inotropic actions, whereas hypothermic intervention elicited positive inotropic actions in cultured cardiomyocytes, isolated trabeculae (papillary muscle), or excised whole hearts, indicating that myocardial temperature directly regulates cardiac contractility^1–7^. The most significant question is why and how myocardial temperature directly regulates cardiac contractility, energy metabolism, and their relationship. Although previous studies reported that the magnitude of Ca^2+^ transient in cardiomyocytes increases in hypothermic intervention^1,6^, it is still unclear whether the amplitude of Ca^2+^ transient decreases in hyperthermic intervention, or its change is really associated with inotropic action and energy metabolism in different thermal interventions.

The alteration of body temperature has been well known to affect cardiac output and myocardial O_2_ consumption by changing cardiac contractility and heart rate. In addition, the alteration of cardiac temperature affects many enzyme activities related to CB cycling, Ca^2+^ handling, and basal metabolism, including energy supply from the mitochondria in cardiomyocytes. However, clarifying the direct effects of changing temperature on cardiac function and energy metabolism using *in vivo* study is difficult. The alteration of thermal condition affects the systemic regulation mediated by neuronal and hormonal factors to regulate not only the cardiac contractility and heart rate, but also the enzyme activities related to CB cycling, Ca^2+^ handling, and basal metabolism.

In the present study, we used the excised, cross-circulated rat heart model to investigate the direct effects of changing temperature on left ventricular (LV) mechanical work and energetics (i.e., mechanoenergetics). Subsequently, we utilized the equivalent maximal elastance (eEmax)–pressure–volume area (PVA)–VO_2_ framework to elucidate the myocardial mechanoenergetics. Previous studies have already reported the effects of changing cardiac temperature on myocardial mechanoenergetics in the excised, cross-circulated canine heart model^3–5,7^. However, canine and rat hearts not only are different in size but also are functionally different. Rodents generally have a much higher heart rate than canines. The LV end-systolic pressure–volume relationship (ESPVR) in canine hearts is linear, whereas that in rat hearts is curvilinear^8–10^. Thus, the direct effects of changing cardiac temperature on myocardial mechanoenergetics have not yet been analyzed in small animal hearts, such as rats.

With this experimental model, we evaluated the LV ESPVR and end-diastolic pressure–volume relationship (EDPVR), and the linear relationship between the myocardial VO_2_ as O_2_ consumption per beat and PVA as a total mechanical energy per beat in isovolumically contracting rat hearts during hypo- (32 °C), normo- (37 °C), and hyperthermia (42 °C) under a 300-beats per minute (bpm) pacing. We aimed to investigate the direct effects of hypo- and hyperthermia, which are likely to be encountered in each lifetime, on LV myocardial mechanoenergetics, using the excised, cross-circulated rat heart model.

## Methods

### Animals

The investigation conformed with the *Guide for the Care and Use of Laboratory Animals* published by the US National Institutes of Health (NIH Publication No. 85-23, revised 1996) and was reviewed and approved by the Animal Care and Use Committee of Nara Medical University and Animal Research Committee of Gifu University. Male Wistar rats weighing 453 ± 67 g were purchased from Japan SLC, Inc. (Hamamatsu, Japan) in the present experiments.

### Excised cross-circulated rat heart model

**Figure 1:**
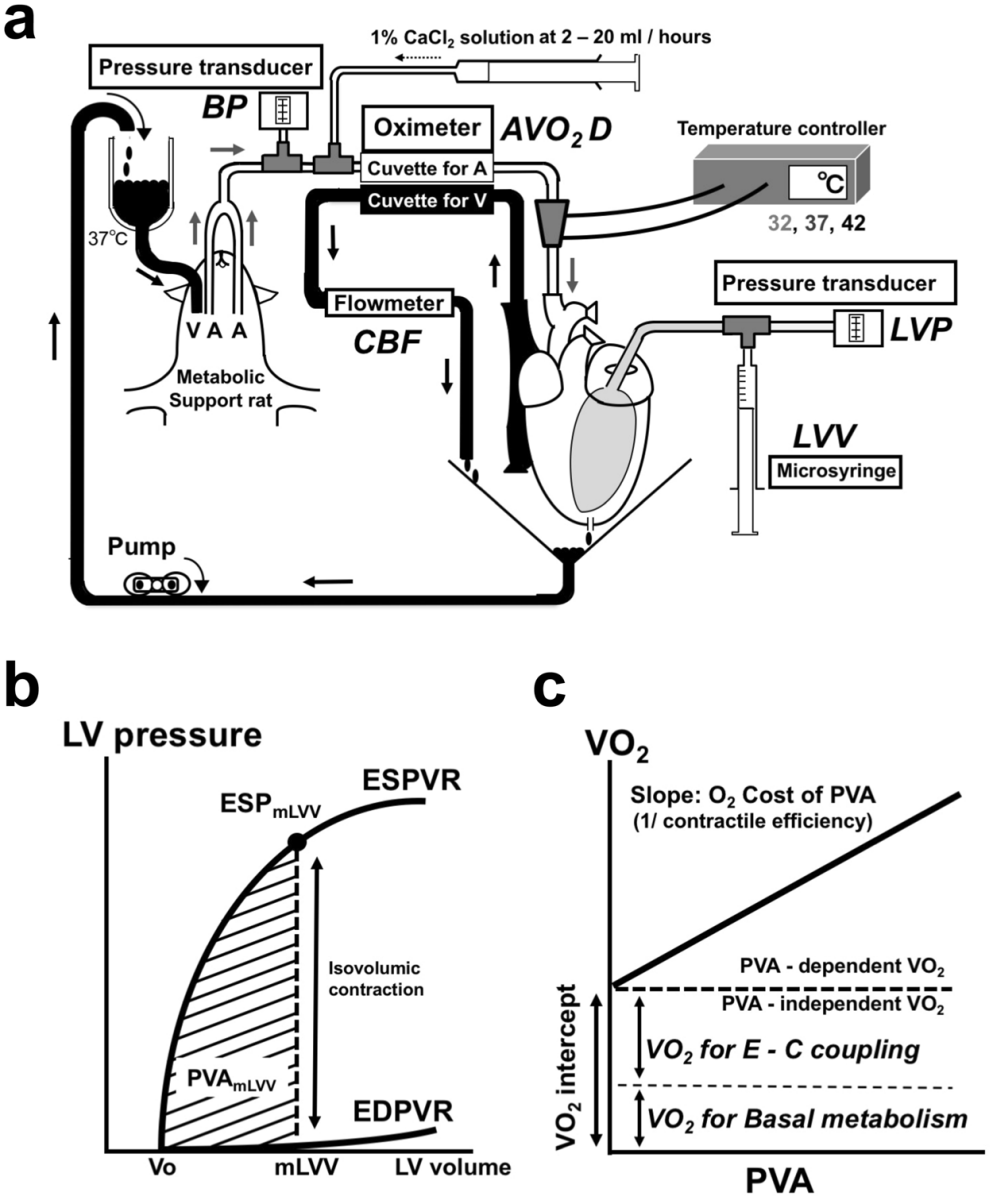
Schematic illustration of experimental setting for the excised blood-perfused rat heart (**a**) and framework of end-systolic pressure–volume relationship (ESPVR)–VO_2_–pressure–volume area (PVA) (**b**,**c**). (**a**) We used three rats in each experiment. The largest one was used as blood supplier. The middle-size one was used as metabolic supporter for the excised heart. The smallest one was used as heart donor in excised cross-circulation rat heart preparation. The perfusion pressure of the excised hearts was maintained at 100 mmHg with controlled blood pressure of the metabolic supporter rats. Total coronary blood flow (CBF) was continuously measured with an ultrasonic flowmeter placed in the middle of the coronary venous drainage tubing from the RV. The coronary arteriovenous O_2_ content difference (AVO_2_D) was continuously measured by passing all arterial and venous cross-circulation blood through the two cuvettes of a custom-made AVO_2_D analyzer. The myocardial temperature was changed from 37 °C to 32 °C or 42 °C with inline-type temperature controller system for pre-incubation 30 min before data sampling. (**b**) LV ESPVR and end-diastolic pressure–volume relationship (EDPVR) at midrange LV volume (mLVV, a half value between the minimum and maximum water volume infused into the balloon). The minimal volume loading LV volume (V_0_) was also determined as the volume-axis intercept of the best-fit ESPVR. The PVA was computed at each LV volume as the area between the ESPVR and the EDPVR and between the V_0_ and the given chamber volume (balloon material volume + intra-balloon water volume). The value of V_0_, mLVV, and PVA were normalized by LV mass to 1 g. A striped area denoted PVA at a mLVV (PVA_mLVV_) (ESP_mLVV_: observed end-systolic pressure at a mLVV). (**c**) Myocardial O_2_ consumption per beat (VO_2_)–PVA relationship. Myocardial VO_2_ was obtained as the product of the CBF and coronary AVO_2_D. The VO_2_–PVA relation was linear in the rat LV. Its slope represents the O_2_ cost of PVA (1/contractile efficiency), and its VO_2_ intercept represents PVA-independent VO_2_. The PVA-independent VO_2_ is composed of O_2_ consumption for Ca^2+^ handling in E-C coupling and for basal metabolism.

We used an excised, cross-circulated rat heart preparation (Fig. 1a) to perform cardiac mechanoenergetics analysis as previously reported in detail^8,11,12^. In brief, the bilateral common carotid arteries and right external jugular vein of the metabolic supporter rat were cannulated and connected to the brachiocephalic artery and right ventricle (RV) via the superior vena cava in the heart donor rat, respectively (Fig. 1a). The beating heart supported by cross-circulation was subsequently excised from the chest of the heart donor rat. The LV epicardial electrocardiogram was recorded, and the heart rate was constantly maintained at 300 beats per minute (bpm) by electrical pacing of the right atrium. A thin latex balloon (balloon material volume, 0.08 ml; corresponding to minimal volume loading LV [V_0_]) fitted into the LV space was connected to a pressure transducer (Life Kit DX-312, Nihon-Kohden, Tokyo, Japan) and to a 0.5-ml precision glass syringe with fine scales (minimum scale: 0.005 ml). The minimal volume loading LV volume (V_0_) was also determined as the volume-axis intercept of the best-fit ESPVR. The perfusion pressure of the excised hearts was maintained at 100 mmHg, with controlled blood pressure of the metabolic supporter rats. Blood lactate was measured with Lactate Pro (Arkray, Kyoto, Japan). We confirmed no increase in the mean values of arteriovenous lactate difference at the maximum LV volume (LVV) loading (the maximum O_2_ demand). In each temperature, lactate production was negligible at the steady state of the maximum LVV loading. Arterial pH, PO_2_, and PCO_2_ of the supporter rat were maintained within their physiological ranges with supplemental O_2_ and sodium bicarbonate. The anesthetic level of the metabolic supporter rat was maintained at a constant level via additional continuous infusion of pentobarbital sodium at 7.5 mg h^−1^ by monitoring the systemic arterial pressure and heart rate.

### Myocardial temperature and experimental protocol

Coronary perfusion of the excised heart was never interrupted during this preparation, and the excised heart was maintained at 37 °C. The myocardial temperature was changed from 37 °C (normothermic condition) to 32 °C (hypothermic condition) or 42 °C (hyperthermic condition) with ThermoClamp™-1 temperature controller system (inline-type, AutoMate Scientific, Inc., CA) for pre-incubation 30 min before data sampling (Fig. 1a). Cooling to 32 °C was performed by incubation at room temperature (21 °C to 26 °C), which was controlled by an air conditioner. LV volume (LVV) was changing and measured by adjusting the intra-balloon water volume with the syringe in 0.025-ml steps between 0.08 ml and 0.23 ml (5 to 6 different volumes) under hypo-, normo-, and hyperthermia (Fig. 1a). The coronary arteriovenous O_2_ content difference (AVO_2_D) was continuously measured by passing all the arterial and venous cross-circulation blood through the cuvettes of a custom made AVO_2_D analyzer (PWA-200S, Shoe Technica; Chiba, Japan) as previously reported in detail^8,11,12^. LV pressure (LVP) and VO_2_ data during isovolumic contractions were simultaneously obtained at each LVV (volume-loading run: vol-run). After the vol-run, a Ca^2+^-induced inotropic run (Ca^2+^ ino-run) was performed at midrange LVV (mLVV) (0.16 ml = 0.08 ml [V_0_] plus 0.08 ml [a half value between the minimum and maximum water volume infused into the balloon]) by intracoronary infusion of 1% CaCl_2_ solution. The infusion rate of Ca^2+^ was increased gradually until we observed a decrease in ESP or arrhythmia due to Ca^2+^ overload. To obtain steady-state data, every data point was measured 3 min after changing the LVV or infusion rate of Ca^2+^. Finally, cardiac arrest was induced by intracoronary infusion of KCl (0.5 mol l^−1^) at 5–10 ml h^−1^ to measure the basal metabolic O_2_ consumption. The VO_2_ and PVA data in KCl-cardiac arrest were obtained at LVV = V_0_ to avoid volume-loading effects. All data were measured and sampled at 1 kHz for 5–10 s and averaged using a PowerLab unit and LabChart software (ADInstruments, Bella Vista, NSW, Australia).

### Analyses of one-beat LV pressure–time curve by logistic function

To evaluate the LV end-diastolic relaxation rate or lusitropism, we analyzed the “logistic” time constant from the respective best-fit functions to one-beat LV pressure–time curve at mLVV during relaxation with our proposed “logistic function”^13^ at 32 °C (n = 10), 37 °C (n = 13), and 42 °C (n = 10).

### Data analysis

We analyzed the obtained data in excised, cross-circulated rat heart preparations as previously reported^8,10–12,14,15^. Briefly, we obtained the best-fit ESPVR and EDPVR from five to six different pressure–volume data with two different exponential functions by means of the least-squares method (DeltaGraph, RedRock Software, Inc., UT, USA) in hypo-, normo-, and hyperthermia. The PVA was calculated as the area surrounded by ESPVR and EDPVR (Fig. 1b). An example of PVA at mLVV in normothermia was shown in Fig. 1b. Myocardial O_2_ consumption per beat (VO_2_) was obtained as the product of coronary blood flow (CBF) and coronary AVO_2_D divided by heart rate (bpm). The VO_2_–PVA relationship was linear in the rat LV (Fig. 1c). Its slope represents the O_2_ cost of PVA, and its VO_2_ intercept represents the PVA-independent VO_2_. Thus, the VO_2_ at given PVA includes PVA-dependent VO_2_ for CB cycling and PVA-independent VO_2_ composed of VO_2_ for Ca^2+^ handling in E-C coupling and basal metabolism (Fig. 1c). Thus, VO_2_ for Ca^2+^ handling in E-C coupling was calculated as PVA-independent VO_2_ minus the basal metabolic VO_2_ obtained by KCl-cardiac arrest at LVV = V_0_.

### O_2_ cost of LV contractility for Ca^2+^

Subsequently, we estimated the O_2_ cost of LV contractility in Ca^2+^ ino-run at each temperature as previously reported^10,12,14–16^. In brief, we fixed LVV at mLVV, and Ca^2+^ (1.0%) was continuously infused into the arterial tube with an infusion pump in hypo-, normo-, and hyperthermia. The VO_2_–PVA linear relationship in the Ca^2+^ inotropism was shifted upward in parallel with the control VO_2_–PVA relationship. The gradually increased VO_2_-intercept values (PVA-independent VO_2_ values) of the lines proportional to the enhanced LV contractility by Ca^2+^ were obtained by this procedure. LV ESPVR is practically linear in large animals, such as canines and humans; therefore, its slope is designated as Emax (i.e., an index for LV contractility)^16^. ESPVR is, however, curvilinear in small animals, such as rats and guinea pigs. Thus, our proposed index for LV contractility, eEmax at mLVV (eEmax_mLVV_), was calculated as the slope of a virtual triangular area equivalent to PVA_mLVV_^10,15^. The O_2_ cost of LV contractility was calculated as the slope of the relationship between PVA-independent VO_2_ and eEmax_mLVV_, which means VO_2_ was used for Ca^2+^ handling in E-C coupling per unit changes in LV contractility. ESP_mLVV_: observed end-systolic pressure at a mLVV

### Immunoblotting analysis for sarcoplasmic reticulum Ca^2+^-ATPase (SERCA) 2, phospholamban (PLB), and phosphorylated-PLB (p-PLB)

Immunoblotting analysis was performed as previously reported^14,17,18^. In brief, total proteins were purified from the LV free wall of each frozen heart stored at −80 °C after the mechanoenergetic studies at each temperature. The proteins (50 µg/lane) were separated on SDS-polyacrylamide gels (10% for SERCA2; 15% for PLB and p-PLB) in a minigel apparatus (Mini-PROTEAN II, Bio-Rad Laboratories, Inc., CA, USA) and transferred to polyvinylidene difluoride membranes. The membranes were blocked (4% Block Ace, Sumitomo Dainippon Pharma Co., Osaka, Japan) and subsequently incubated with primary antibody against anti-SERCA2 antibody (1:1000 dilution, Thermo Fisher Scientific Inc., IL, USA), anti-PLB antibody (1:1000 dilution, Upstate Biotechnology, Inc., MA, USA), and anti-p-PLB (Ser16) antibody (1:1000 dilution, Upstate Biotechnology, Inc., NY, USA). The detection was performed using the luminescence method (ECL western blotting detection kit, GE Healthcare Japan, Tokyo, Japan) with peroxidase-linked anti-mouse IgG (1:5000 dilution) or peroxidase-linked anti-rabbit IgG (1:5000). The bands were normalized to anti-GAPDH antibody (Cell Signaling Technology Inc., MA, USA) to confirm equal loading of samples. Band intensity was analyzed using ImageJ 1.43 software.

### Statistics

We compared the VO_2_–PVA regression lines of the vol-runs at three different temperatures in each heart by analysis of covariance (ANCOVA). ANCOVA was also used to compare the regression lines of PVA–independent VO_2_ on LV contractility (eEmax) among the three temperatures. Multiple comparisons were performed using one-way ANOVA with post-hoc Bonferroni test. A value of p < 0.05 was considered statistically significant. All data were expressed as mean ± SD.

### Ethical approval

All applicable international, national, and/or institutional guidelines for the care and use of animals were followed. The investigation conformed with the *Guide for the Care and Use of Laboratory Animals* published by the US National Institutes of Health (NIH Publication No. 85-23, revised 1996) and was reviewed and approved by the Animal Care and Use Committee of Nara Medical University and Animal Research Committee of Gifu University.

## Results

### Effects of cardiac thermal conditions on lusitropy of the heart

**Figure 2:**
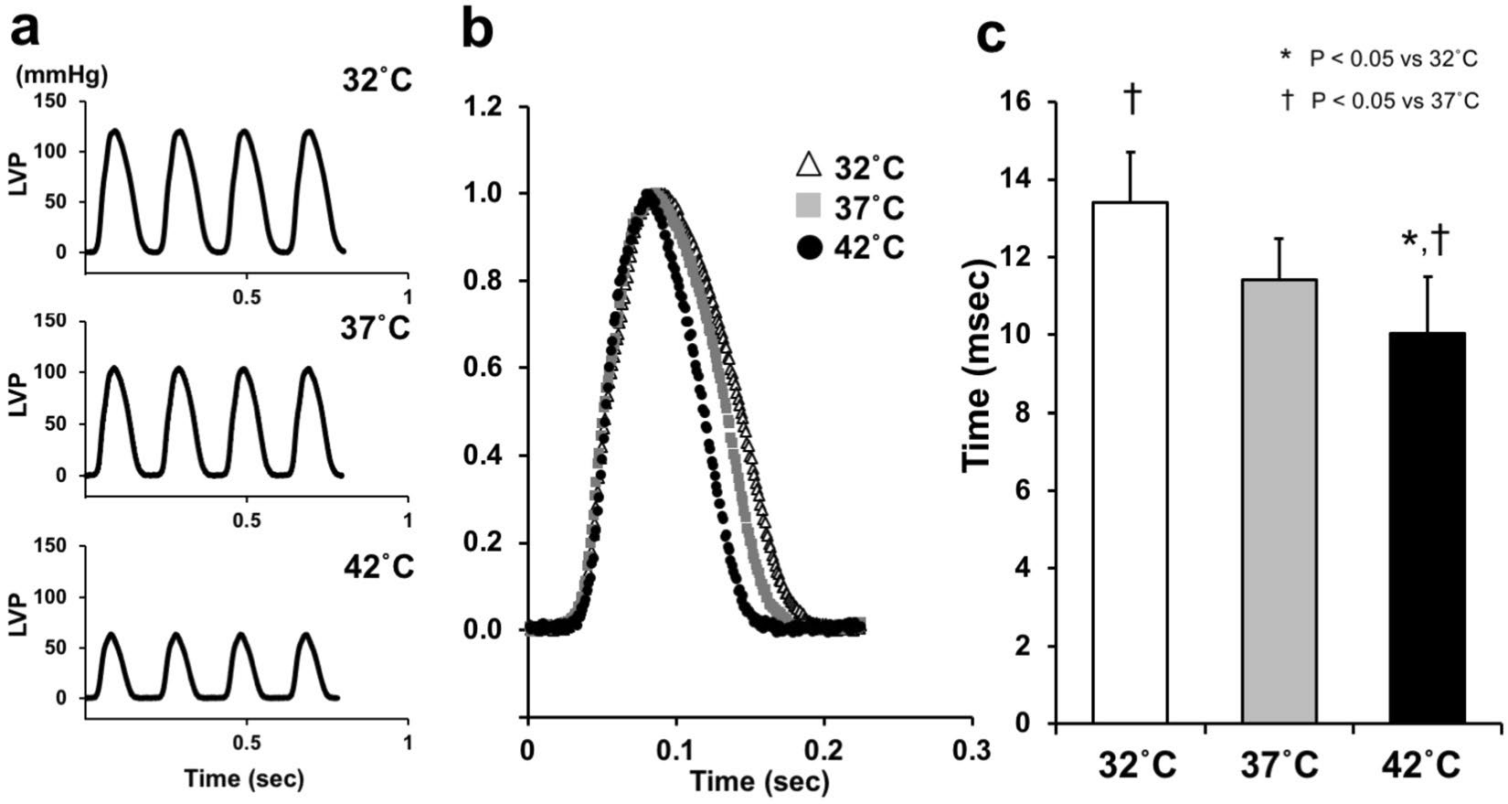
Representative data of LV pressure–time curves (**a**), normalized LV pressure (LVP)–time curves (**b**), and comparison of mean logistic time constants (**c**) at mLVV at 32 °C (hypothermia, n = 10), 37 °C (normothermia, n = 13), and 42 °C (hyperthermia, n = 10). These data obviously showed decreased maximal LVP and reduced duration of LV relaxation time with increasing cardiac temperature. Values are presented as means ± SD. *p < *0.05 vs. 32* *°C*, ^†^*p* < *0.05 vs. 37* *°C.*

First, we analyzed the effects of changing cardiac thermal conditions on pressure–time curves in the same heart. Figure 2a shows that ESPs_mLVV_ (infused volume 0.08 ml) had a marked decrease that is associated with increasing temperature in the heart.

To reveal changes in the lusitropy of LV associated with changing cardiac temperatures, we compared normalized LV pressure–time curves and logistic time constants^13^. The relaxation time in hyperthermia was shorter than that in normothermia, and the relaxation time in hypothermia was longer than that in normothermia (Fig. 2b). The mean logistic time constant significantly shortened in hyperthermia and lengthened in hypothermia compared with normothermia (both p < 0.05) (Fig. 2c). Furthermore, the mean logistic time constant in hyperthermia significantly shortened than that in hypothermia (p < 0.05) (Fig. 2c). These results suggested that the increasing cardiac temperature improved diastolic function in the hearts.

### Effects of cardiac thermal conditions on ESPVRs and VO_2_–PVA relationships of the heart

**Figure 3:**
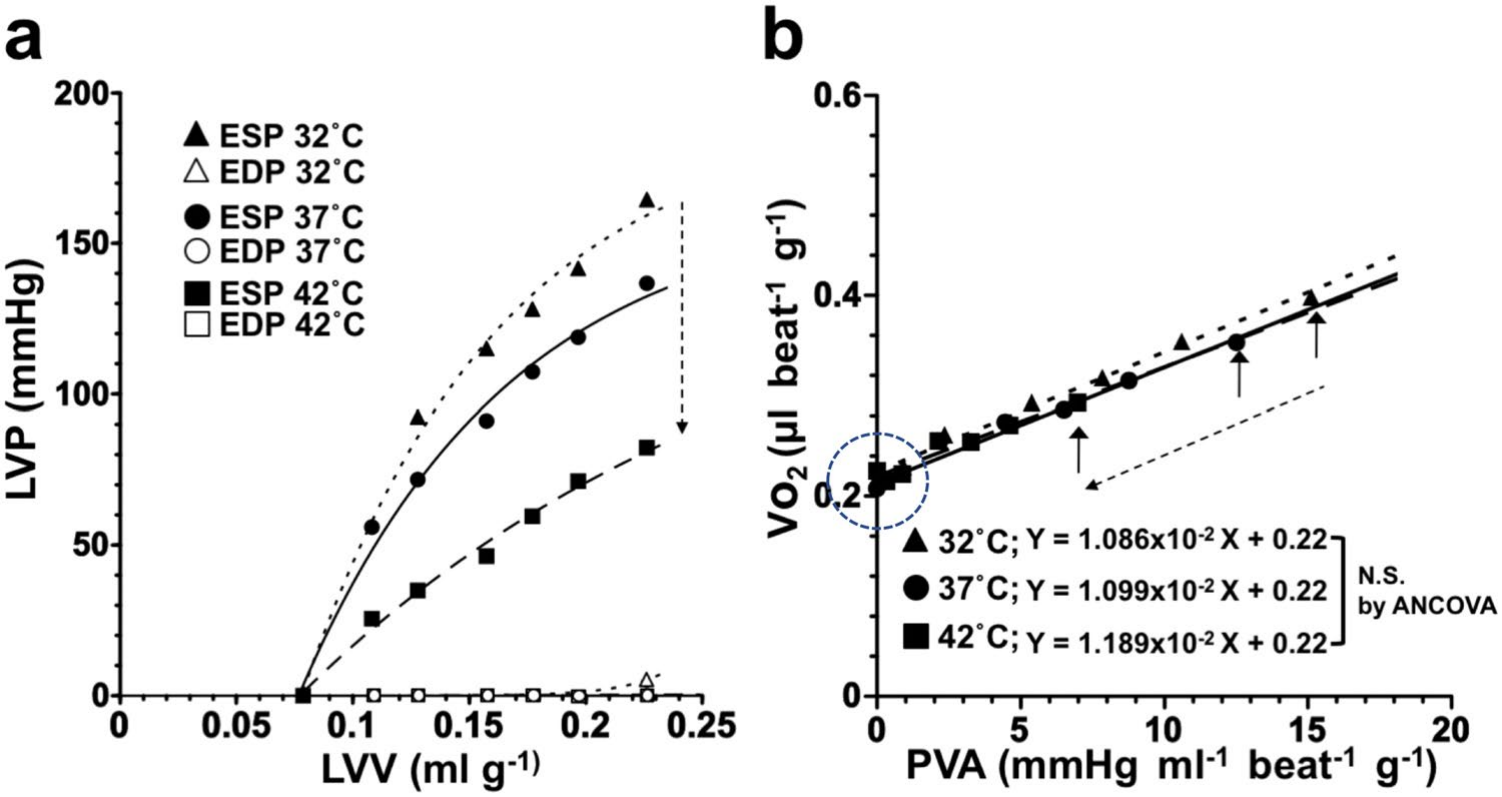
Representative data of LV ESPVR and EDPVR (**a**), and VO_2_–PVA relationship (**b**) at 32 °C (hypothermia, n = 10), 37 °C (normothermia, n = 13), and 42 °C (hyperthermia, n = 10). The ESPs and EDPs data were recorded at each fixed LV volume in hypo-, normo-, and hyperthermia. (**a**) As shown by a dashed long arrow in the left panel, LV ESPVR shifted downward (**a**), and LV EDPVR ained unchanged, but VO_2_–PVA relationship could be superimposed; neither slope nor VO_2_ intercept changed (**b**) with increasing cardiac temperature. Thus, each data point of VO_2_–PVA at each LVV shifted left-downward with increasing cardiac temperature (a dashed long arrow in the right panel) from hypothermia (solid triangle) to hyperthermia (solid square) passing through normothermia (solid circle) on the superimposable VO_2_–PVA relationship line. As shown by a dashed open circle in the right panel (b), unchanged VO_2_ intercepts indicate that PVA-independent VO_2_ were not affected by changing cardiac temperature.

Representative data of ESPVRs and EDPVRs in an identical heart during hypo-, normo-, and hyperthermia are shown in Fig. 3a. ESPVRs shifted downward with increasing cardiac temperature, as indicated by a dashed arrow (Fig. 3a). The mean ESPs_mLVV_ and PVAs_mLVV_ significantly decreased when the cardiac temperature increased to 42 °C (p < 0.05) (Table 1). EDPVRs did not change at these thermal conditions. These results suggested that the increasing cardiac temperature showed negative inotropic action. In contrast, the decreasing cardiac temperature showed positive inotropic action in the present study.

**Table 1:** Mean variables of left ventricular (LV) mechanics.

	32 °C (n = 10)	37 °C (n = 13)	42 °C (n = 10)
A (mmHg)	221.7 ± 25.3	212.7 ± 30.9	191.6 ± 52.3
B (ml^−1^)	13.3 ± 7.00	10.3 ± 2.90	8.3 ± 3.8^*^
V_0_ (ml g^−1^)	0.090 ± 0.012	0.096 ± 0.016	0.091 ± 0.013
mLVV (ml g^−1^)	0.167 ± 0.027	0.174 ± 0.029	0.177 ± 0.031
ESP_mLVV_ (mmHg)	134.6 ± 17.0^†^	103.4 ± 10.7	76.0 ± 11.5^*,†^
PVA_mLVV_ (mmHg ml^−1^ beat^−1^ g^−1^)	6.93 ± 1.46^†^	5.59 ± 1.46	4.11 ± 1.13^*,†^

All values are means ± SD. ^*^p < 0.05 vs 32 °C, ^†^p < 0.05 vs 37 °C. A and B, parameters of best-fit end systolic pressure (ESP)-volume relation (ESPVR) obtained by the equation: A{1 − exp[−B(V − V_0_)]}. V_0_, volume intercept of ESPVR. mLVV, left ventricular volume at midrange. ESP_mLVV_, ESP at mLVV. PVA_mLVV_, pressure-volume area at mLVV.

**Figure 4:**
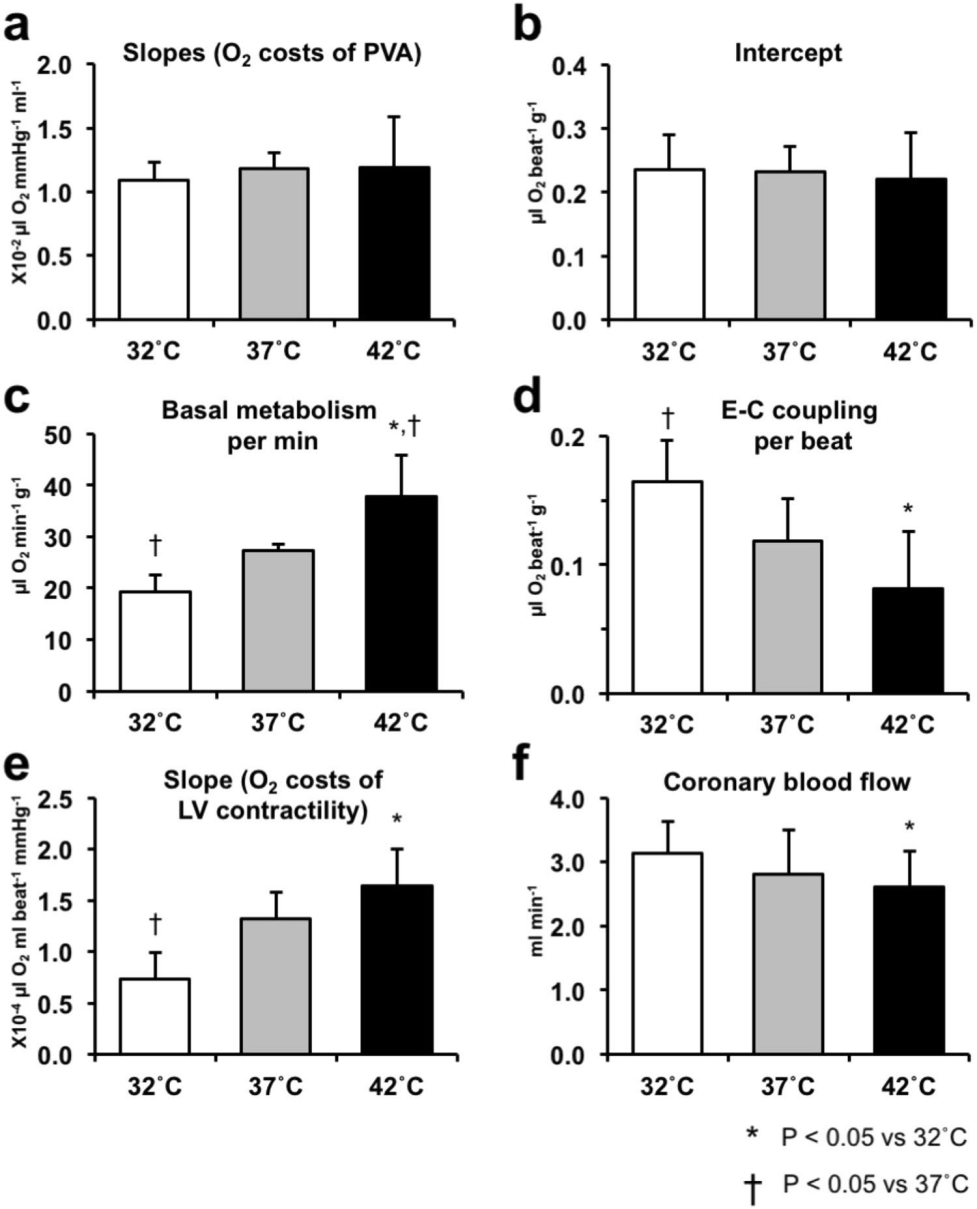
Comparison of the mean slopes (O_2_ costs of PVA) (**a**), VO_2_ intercepts (PVA independent VO_2_) (**b**), O_2_ consumption for basal metabolism per minute (**c**), VO_2_ for excitation–contraction (E-C) coupling (**d**), O_2_ costs of LV contractility for Ca^2+^ (**e**), and CBF (**f**) at 32 °C (hypothermia, n = 10), 37 °C (normothermia, n = 13), and 42 °C (hyperthermia, n = 10). CBF in hyperthermia is significantly smaller than that in hypothermia, which may be related to lower LV contractility (LVV = 0.16, balloon material volume = 0.08 ml plus balloon water volume = 0.08 ml). We confirmed no lactate production in the heart through the experiments, indicating ischemia was not induced. Values are presented as means ± SD. *p < *0.05 vs. 32* *°C*, ^†^*p* < *0.05 vs. 37* *°C.*

Figure 3b shows the three superimposable VO_2_–PVA linear relationships in this heart. Each data point (indicated by three vertical arrows) of each VO_2_–PVA relationship at the same LVV (infusion volume = 0.15 ml) shifted left-downward from hypothermia (indicated by a solid triangle) to hyperthermia (indicated by a solid square), passing through normothermia (indicated by a solid circle), although their intercepts and slopes remained unchanged. These results indicated unchanged PVA-independent VO_2_ (a dashed open circle on the VO_2_–axis) and decreased PVA-dependent VO_2_ (a dashed long arrow), which was associated with increasing cardiac temperature. The latter PVA-dependent VO_2_ decrease is considered to be caused by inhibition of CB cycling. The mean data obtained from VO_2_–PVA relationships (n = 10 in hypothermia, n = 13 in normothermia, n = 10 in hyperthermia) showed that their slopes and VO_2_ intercepts did not change, but VO_2_ intercept composition elements (i.e., basal metabolic O_2_ consumption) significantly increased (Q_10_ = 1.96, data not shown) (p < 0.05), and the VO_2_ for E-C coupling decreased instead, which was associated with increasing the cardiac temperature to 42 °C (p < 0.05) (Fig. 4a–d).

CBF significantly decreased, which was associated with increasing the cardiac temperature to 42 °C (p < 0.05, vs. 32 °C) (Fig. 4f).

### Effects of cardiac thermal conditions on the O_2_ cost of LV contractility of the hearts

**Figure 5:**
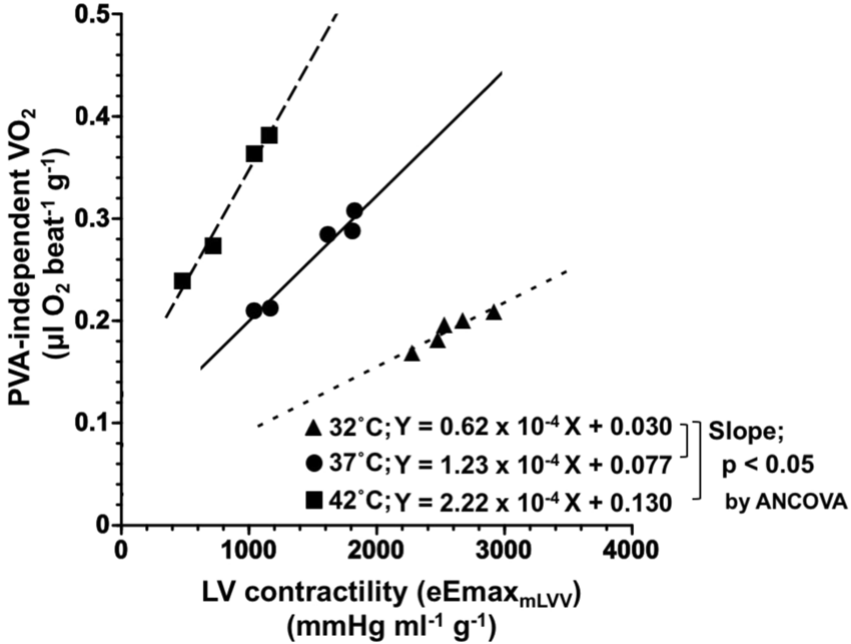
Representative PVA-independent VO_2_-equivalent maximal elastance (eEmax) at mLVV (eEmax_mLVV_) relationships at 32 °C (hypothermia, close triangle), 37 °C (normothermia, close circle), and 42 °C (hyperthermia, close square). The slope of PVA-independent VO_2_–eEmax_mLVV_ relationships indicates the O_2_ cost of LV contractility for Ca^2+^. The values are presented as mean ± SD, which are shown in Fig. 3e.

Figure 5 shows the representative relationships between VO_2_ for Ca^2+^ handling in E-C coupling and eEmax_mLVV_ during Ca^2+^ inotropism run under hypo-, normo-, and hyperthermia. These three distinct linear relationships in each thermal condition had different slopes, which meant that the O_2_ costs of LV contractility were different. The O_2_ cost of LV contractility for Ca^2+^ was significantly increased, which was associated with increasing cardiac temperature (p < 0.05, vs. 32 °C) (Fig. 4e). These results indicate that the O_2_ cost of LV contractility for Ca^2+^ increased with increasing cardiac temperature.

### Effects of cardiac thermal conditions on the amounts of Ca^2+^ handling proteins of the hearts

**Figure 6:**
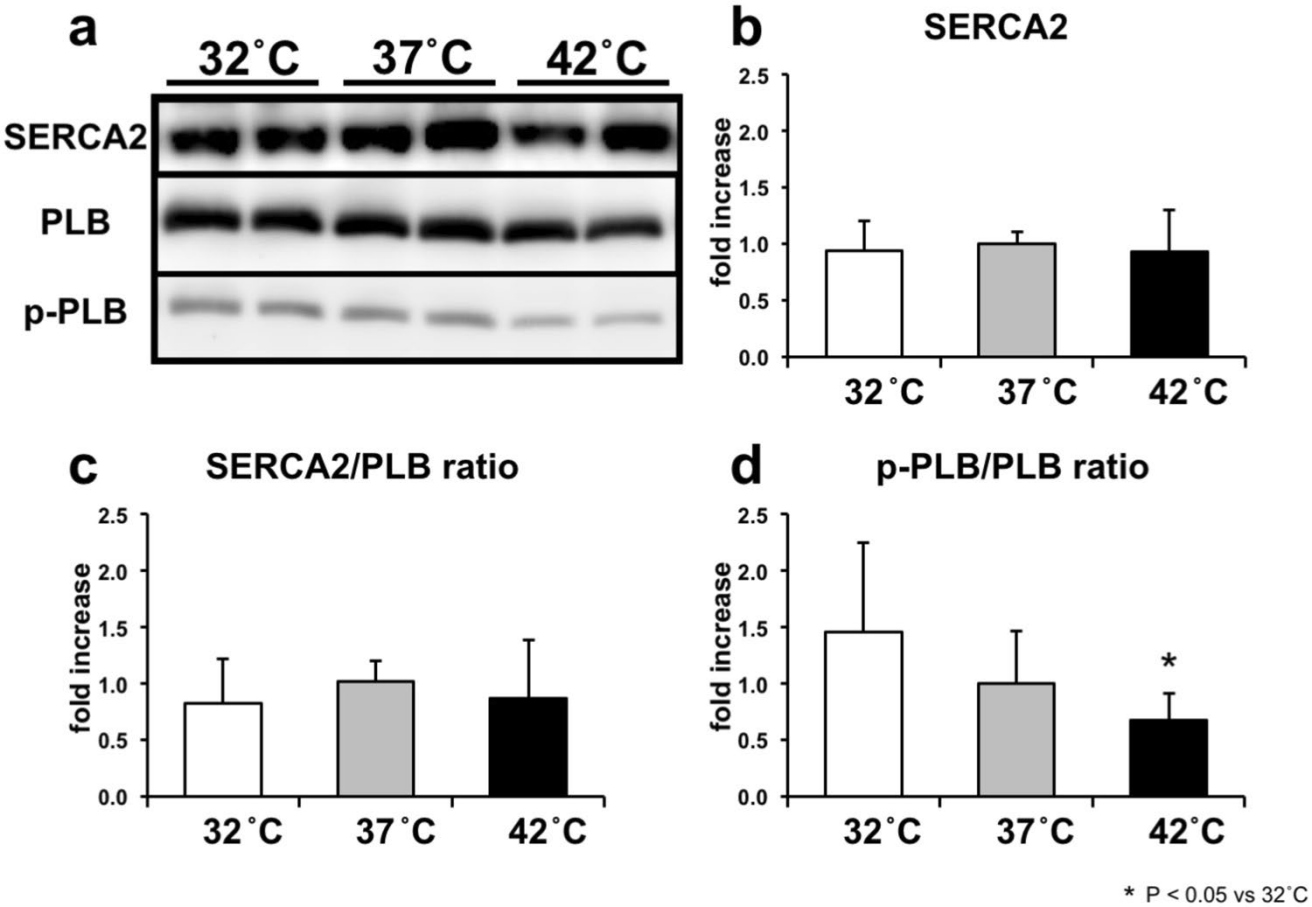
Western blot analysis of SERCA2, phospholamban (PLB), and phosphorylated phospholamban (p-PLB) in LV tissues of hypo- (n = 5), normo- (n = 7), and hyperthermia (n = 5). Representative data of SERCA2, PLB, and p-PLB. (**a**) Comparison of the mean protein levels of SERCA2 (**b**) and the ratios of SERCA2/PLB (**c**) and p-PLB/ PLB (**d**). Values are means ± SD. *p < *0.05 vs. 32* *°C.*

We examined the levels of Ca^2+^ handling proteins, SERCA2, PLB, p-PLB, and GAPDH as an internal control in the LV tissues during hypo-, normo-, and hyperthermia. The ratio of p-PLB/PLB was significantly decreased, which was associated with increasing the cardiac temperature to 42 °C (p < 0.05, vs. 32 °C) without changing the amounts of SERCA2 and PLB proteins, and SERCA2/PLB ratio. The results indicate that increasing the cardiac temperature contributes to SERCA2 activity inhibition, and thus, suppress myocardial Ca^2+^ handling in E-C coupling (Fig. 6).

## Discussion

**Figure 7:**
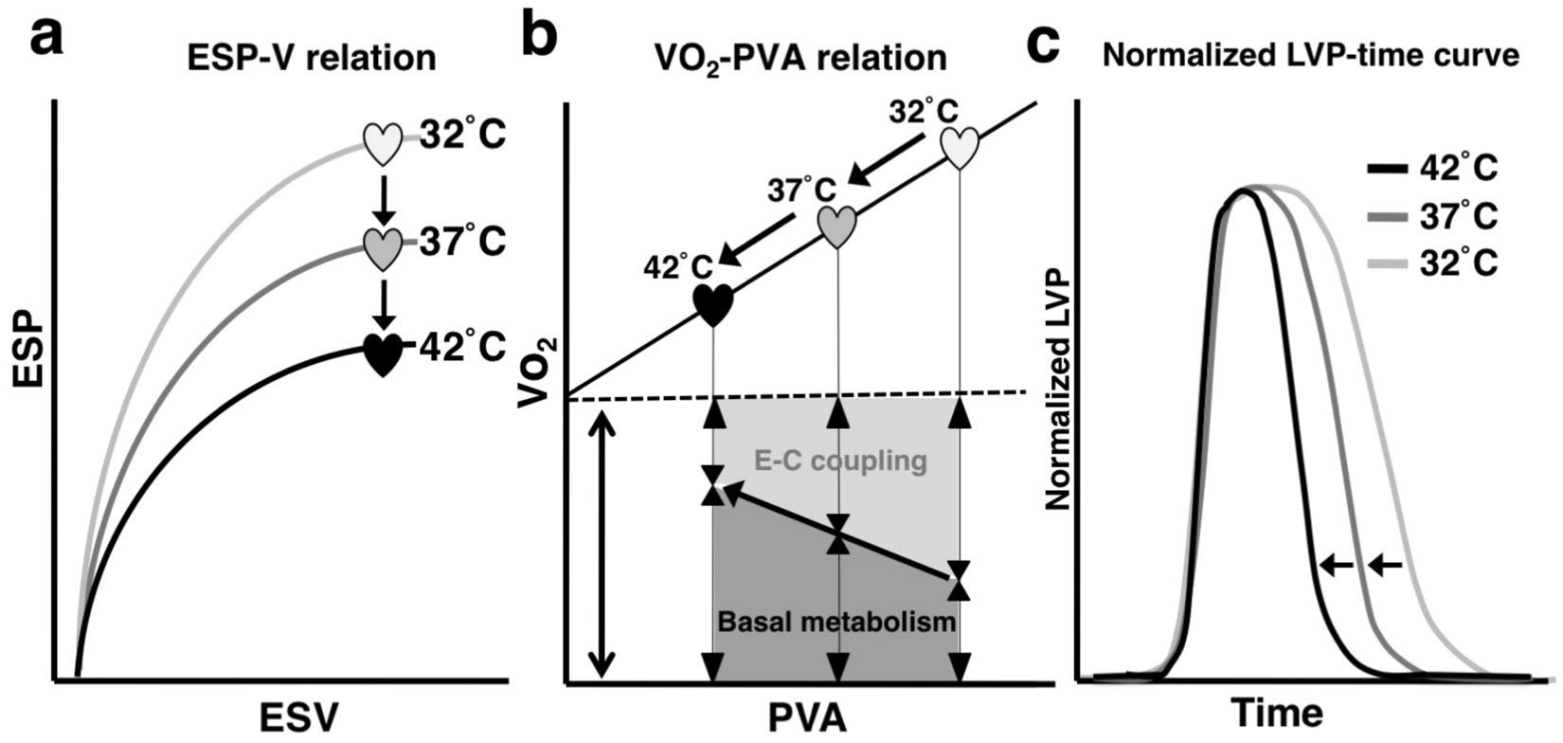
Schematic illustrations of ESPVR (**a**), VO_2_–PVA relationship (**b**), and normalized LVP–time curves (**c**) on cardiac mechanoenergetics in hypo-, normo-, and hyperthermia.

The findings of the present study with increasing the cardiac temperature from at least 32 °C to 42 °C at a fixed pacing heart rate are that^19^ LV ESPVR shifted downward^20^. The slopes and VO_2_ intercepts of VO_2_–PVA linear relationships did not change under these thermal conditions, indicating the unchanged O_2_ cost of PVA (i.e., transduction efficiency from ATP to mechanical work)^8^. However, the VO_2_–PVA data point at each preload (LVV) shifted left-downward on the superimposable VO_2_–PVA lines^11^. The O_2_ consumption for basal metabolism increased, and the VO_2_ for E-C coupling reversely decreased, although the summation of both was unchanged^1^. The O_2_ cost of LV contractility for Ca^2+^ increased, indicating that more O_2_ is needed to exert the same LV contractility^21^.The ratio of p-PLB/PLB decreased without changing the amounts of SERCA2 and PLB proteins, indicating SERCA2 activity inhibition. Accordingly, the VO_2_ for Ca^2+^ handling in E-C coupling decreased^22^. The logistic time constants evaluating for LV relaxation time were significantly shortened with increasing cardiac temperature. Finally, we summarized the major findings of the present study in Fig. 7.

The ESPs_mLVV_ and PVAs_mLVV_ obviously decreased, and LV ESPVRs shifted downward, indicating a negative inotropic action with increasing cardiac temperature (Table 1 and Fig. 7a). Previous studies also reported that increasing the cardiac temperature from at least 30 °C to 42 °C showed a negative inotropic action in whole canine hearts and decreased force development in cardiac isolated rat trabeculae^1,4,5^. A possible mechanism for the negative inotropic action with increasing cardiac temperature has been suggested to accelerate the rate of detachment in CB cycling and/or decreasing the number of myosin heads interacting with the thin filament (actin) due to increased myosin ATPase activity.

In contrast, decreasing the cardiac temperature prolonged the duration of myocardial contraction and increased the force development by decreasing CB detachment rates in isolated rabbit or rat papillary muscles^1,23,24^. In addition, cooling increases Ca^2+^ sensitivity of troponin C (TnC) in intact rabbit hearts^3,25^. These previous reports suggest the underlying mechanisms for the positive inotropic action (Fig. 7a) and lengthening of LV relaxation time (Fig. 7c) in hypothermia. In contrast, we can consider that the faster relaxation during hyperthermia as shown by Fig. 7c would be due to acceleration of the detachment of CB cycling rather than acceleration of Ca^2+^ uptake to sarcoplasmic reticulum (SR) via SERCA.

Changing the cardiac temperature did not change the slopes and VO_2_ intercepts of the VO_2_–PVA relationships although positive and negative inotropic actions in hypothermia and hyperthermia occurred, respectively. Many previous studies, including our study, showed that VO_2_ intercepts increased and decreased in rats and canines treated with positive and negative inotropic drugs, respectively, without changing the slopes of the VO_2_–PVA relationships (i.e., parallel shifts of VO_2_–PVA linear relationships caused by the positive or negative inotropic drugs are associated with the increase or decrease in Ca^2+^ handling VO_2_ in E-C coupling)^20,22,26–30^. Our recent study showed a significantly smaller mean slope of VO_2_–PVA relationships in cardiac SERCA2a-overexpressed transgenic rats than that in wild-type rats^18^. The present study showing an unchanged slope indicated that the efficiency of chemo-mechanical energy transduction was maintained even if the contribution of SERCA activity in E-C coupling was changed by thermal intervention.

The most interesting finding of the present study is that the VO_2_ for E-C coupling decreased, but the O_2_ consumption in basal metabolism increased despite the unchanged VO_2_ intercepts with increasing cardiac temperature (Fig. 7b). The VO_2_ for Ca^2+^ handling in E-C coupling is mainly consumed by SERCA that transfers Ca^2+^ from the cytosol to the SR at the expense of ATP hydrolysis during muscular relaxation. Therefore, hyperthermic conditions decreased the VO_2_ for Ca^2+^ handling in E-C coupling (Fig. 7b) by the decrease in SERCA activity for Ca^2+^ handling (Fig. 6d). It is unclear whether the reduction of Ca^2+^ release from SR induces the negative inotropic effect during hyperthermia in the present study. Previous studies demonstrated that the magnitude of Ca^2+^ transient in cardiomyocytes increases in hypothermic intervention^1,6^. In the present study, we showed the decrease of p-PLB and VO_2_ for Ca^2+^ handling in E-C coupling in hyperthermia, which indicates the suppression of Ca^2+^ uptake into SR by SERCA. Thus, it is reasonable to suppose that SR Ca^2+^ loading is modulated by cardiac thermal conditions. This is the first report showing that elevating cardiac temperature directly decreased the phosphorylation of PLB without affecting neuro-, and/or humoral factors. In contrast, hypothermic conditions increased the VO_2_ for Ca^2+^ handling in E-C coupling (Fig. 7b), which does not arise from the increase in SERCA activity for Ca^2+^ handling (Fig. 6d), although the amplitude of Ca^2+^ transient may be elevated by increased SR Ca^2+^ release^31,32^.

The O_2_ consumption for basal metabolism increased during increasing cardiac temperature at a Q_10_ of 1.96 despite the unchanged VO_2_ intercepts (Fig. 7b). Previous studies reported an increase in O_2_ consumption for myocardial basal metabolism during temperature increase at a Q_10_ of 1.1 to 1.4 in canine hearts^4,27^. The value of Q_10_ in rat hearts was higher than that in canine hearts, indicating that small animals, such as rats, tend to be more affected by the alteration of cardiac temperature^33^.

Furthermore, the O_2_ cost of LV contractility for Ca^2+^ increased, which was associated with increasing cardiac temperature during decreased SERCA2 activity. The underlying mechanism for this could be suggested that the increased intracellular Ca^2+^ with Ca^2+^ loading is dominantly removed from the cytosol via Na^+^/Ca^2+^ exchanger (NCX), rather than Ca^2+^ uptake via the SERCA2a to SR. This postulate was supported by previous studies reporting that the NCX current and contribution of NCX as Ca^2+^ transporters are highly temperature dependent in guinea pig ventricular myocytes^21,34^.

Although NCX per se does not consume ATP to remove cytosolic Ca^2+^ in exchange with Na^+^ influx (stoichiometry of 3Na^+^:1Ca^2+^), Na^+^ influx must be pumped out by Na^+^/K^+^-ATPase, with a stoichiometry of 3Na^+^:2 K^+^:1ATP, resulting in the net stoichiometry of 1Ca^2+^:1ATP. In contrast, SERCA2a removes cytosolic Ca^2+^ based on a stoichiometry of 2Ca^2+^:1ATP^10^. The O_2_ cost of LV contractility for Ca^2+^ is determined by myofilament Ca^2+^ responsiveness (i.e., the LV contractility/Ca^2+^ handling and/or the Ca^2+^ handling/ATP ratio in the SR)^10,15,16^. Therefore, the O_2_ cost of LV contractility in hyperthermia is higher than that in hypothermia (Figs 4e and 5). On the other hand, VO_2_ for Ca^2+^ handling in E-C coupling simply decreased (Fig. 7b). The above mechanism seems not to work at minimal volume loading LV [V_0_] without Ca^2+^ infusion (see *Methods*) because intracellular Ca^2+^ can be adequately removed from cytosol to SR even though SERCA activity moderately (approximately 67% of normothermia) decreased.

As previously mentioned, cooling increases Ca^2+^ sensitivity of TnC^3,25^ and/or decreases the rates of CB detachment in cardiomyocytes^1,23,24^. Consequent acceleration of myofilament Ca^2+^ responsiveness may decrease the O_2_ cost of LV contractility for Ca^2+^ in hypothermia.

The secondary changes in cardiac contractility and heart rate generated by increasing or decreasing the cardiac temperature would modify the primary changes in cardiac mechanoenergetics of an *in situ* beating heart, where the neural and hormonal factors are influenced by body temperature. Consequently, the cardiac contractility and heart rate would be affected by neural and hormonal factors. However, in the present study, we used 300 bpm with electrical pacing. Therefore, we could exclude the possibility for any interferences from secondary changes in heart rate.

Previous studies in the excised, cross-circulated canine heart model maintained a constant heart rate at 120 bpm^3–5,7^. These studies demonstrated that the ESPVR shifted downward, VO_2_ for E-C coupling decreased, and O_2_ consumption for basal metabolism increased proportionally when the cardiac temperature was increased from 30 °C to 40 °C, whereas PVA-independent VO_2_ and O_2_ cost of PVA remained unchanged. Present results are in accordance with these previous results, indicating that the effects of thermal conditions on cardiac contractility and energy metabolism are common among small and large animal hearts despite of different heart rate, myosin isozyme and size. However, previous studies using canine hearts^4,5^ did not analyze the underlying mechanism for these effects. They only speculated that the mechanism for the effects of different thermal conditions would be related to either Ca^2+^ responsiveness of TnC in CB cycling or modulation of Ca^2+^ handling in E-C coupling, or both^3–5,7^. In contrast, we have obtained the first evidence showing the decrease of VO_2_ for Ca^2+^ handling in E-C coupling is caused by the suppression of SERCA activity due to phospholamban phosphorylation inhibition.

In the present study, used myocardial temperatures were 32 °C, 37 °C, and 42 °C as three different thermal intervention. We found that increasing the cardiac temperature during 32 °C to 42 °C induced negative inotropic action, which was related to increased myosin ATPase activity in CB cycling and inhibited SERCA activity via the declined phosphorylation of PLB in Ca^2+^ handling, respectively. Instead, O_2_ consumption for basal metabolism increased with unchanged VO_2_ intercepts of VO_2_–PVA relationships. The communication between SR and the mitochondria to coordinate energy production and intracellular Ca^2+^ control is well known^19,35^. Therefore, any modulatory interactions between the mitochondria and SR might contribute to the unchanged VO_2_ intercepts.

It remains unknown how the heart senses the thermal condition and transmits the information to signal transduction mechanism, such as phosphorylation of PLB. The transient receptor potential (TRP) channel families may work as micro-thermosensors in cardiomyocytes. In fact, we previously reported that high-dose capsaicin, as TRPV1 agonist, induced direct negative inotropic action on cardiac muscles^36^. Furthermore, we have found that capsazepine, as TRPV1 antagonist, inhibits the hyperthermia-induced negative inotropic action (unpublished observation).

Finally, we concluded that the modulatory interactions among CB cycling, Ca^2+^ handling, and basal metabolism underlie positive or negative inotropism in hypo- or hyperthermic rat hearts, with unchanged efficiency for converting chemical energy into mechanical work.
